# Anaerobiosis, a neglected factor in phage-bacteria interactions

**DOI:** 10.1128/aem.01491-23

**Published:** 2023-11-15

**Authors:** Santiago Hernández Villamizar, Luis A. Chica Cárdenas, Laura T. Morales Mancera, Martha J. Vives Florez

**Affiliations:** 1 Department of Biological Sciences, Universidad de los Andes, Bogotá, Colombia; University of Nebraska-Lincoln, Lincoln, Nebraska, USA

**Keywords:** bacteriophages, *Salmonella*, anaerobiosis, molecular biology, transcriptomic

## Abstract

Phage therapy is a promising alternative in treating bacterial infections; however, understanding how bacteria and phages interact according to the environment is crucial. Although most phage therapy applications are intended for anaerobic environments (such as intestine or wounds), research on the effects of oxygen absence on the phage infection process is scarce . Here, we studied the effect of anoxic conditions in a *Salmonella* sp. – phage model, given the prevalence of this bacterial pathogen in anaerobic environments, and the potential of phage therapy for its control. Our results show that oxygen-deprived conditions affect the life cycle length of the phage and its capacity to control the bacterial population. Changes in host cell size associated with the FtsZ protein were observed, along with a modified emergence of resistance against the phage under different oxygen conditions. Interestingly, transcriptomic analyses revealed that phage infection in aerobic conditions triggers the expression of genes associated with nitrate reduction, an alternative pathway for anaerobic respiration. Meanwhile, uninfected anaerobic cultures displayed the upregulation of genes involved in a different anaerobic metabolic route comprising the transport of thiosulfate and sulfate. Altogether, our results provide insights into the importance of considering oxygen availability as an important factor for phage therapy when working with facultative anaerobic bacteria.

**IMPORTANCE:**

Many parameters affect phage-bacteria interaction. Some of these parameters depend on the environment in which the bacteria are present. Anaerobiosis effect on phage infection in facultative anaerobic bacteria has not yet been studied. The absence of oxygen triggers metabolic changes in facultative bacteria and this affects phage infection and viral life cycle. Understanding how an anaerobic environment can alter the behavior of phages during infection is relevant for the phage therapy success.

## INTRODUCTION

Bacteria inhabit diverse environments and play key roles in various processes, some of which are exclusive to anaerobic habitats (1). Organisms with anaerobic metabolisms can be categorized as strict or facultative, thriving in environments like sludge, ocean depths, and animal intestines where oxygen levels are limited. Bacteriophages, also known as phages, viruses that solely infect bacteria and constitute the most abundant biological entities on Earth (2), exert control over bacterial populations, indirectly influencing a wide array of bacterial-associated processes (3–6). The bulk of our understanding of phage infections and viral cycles has been derived from aerobic conditions using aerobic or facultative bacteria. Only a handful of research groups working with obligate anaerobic bacteria, like *Clostridium* spp. (7), *Bacteroides* spp. (8–10), or *Desulfovibrio* spp. (11, 12), have managed to isolate and characterize their phages. However, due to its lethal impact on strictly anaerobic bacteria, the role of oxygen remains elusive in these models. Consequently, the effect of oxygen concentration on host-phage interactions largely remains an enigma.

Considering the profound dependence of phages on their hosts, any shifts in bacterial metabolic status should correspondingly alter phage infection dynamics and viral replication. For instance, Yamamoto et al. demonstrated that under aerobic growth conditions, the chromosomal DNA (cDNA) of *Salmonella* Typhimurium relaxed, while it remained supercoiled under anaerobic conditions, leading to distinct gene expressions in *topI* (DNA topoisomerase I) and *gyrA* (DNA gyrase subunit A). This suggests the regulation of genes crucial for growth in aerobic or anaerobic environments (13). Schiemann and Shope provided another illustration of the potential impacts of anaerobiosis, showcasing that *S*. Typhimurium *in vitro* growth under anaerobic conditions led to increased bacterial cell uptake by Henle 407 epithelial and mouse peritoneal cells, along with repression of at least one major outer membrane protein (14).

It has been reported that in facultative anaerobic bacteria, ArcA and fumarate and nitrate reductase regulatory (FNR) systems regulate anaerobic metabolism (15–18). FNR serves as a transcriptional regulator, influencing the expression of BtuB protein in *Salmonella* under anaerobic conditions (17). BtuB, an outer membrane protein, plays a role in transporting cobalamins (such as vitamin B12) into the cell. Interestingly, *Salmonella* synthesizes vitamin B12 only under anaerobic conditions (19–21). Given that BtuB acts as a phage receptor in enteric bacteria, including *Salmonella* (22–26), we hypothesize that phage infection could be impacted if BtuB levels decrease during anaerobic growth, as cells would be producing their own vitamin B12 and potentially require less from the growth medium.

Nutrient availability also exerts influence over bacterial physiology. Numerous experiments have demonstrated a direct correlation between cell size and nutrient sources and growth rates (27). For instance, when cultured in a nutrient-rich medium, *Salmonella* cells are roughly twice the size of those cultured in a nutrient-poor environment (28). Additionally, the quantity of FtsZ protein within cells correlates with cell size. Elevated FtsZ levels result in minicell formation, while lower levels delay cell division, yielding larger cells (29, 30).

In our study, we employed *Salmonella* s25pp and its phage ϕSan23 as a model to investigate phage infections within a facultative bacterial species under strictly anaerobic conditions. Utilizing reverse transcription quantitative real-time PCR (RT-qPCR), Western blot, and RNA-seq, we aimed to elucidate and compare gene expression under both aerobic and anaerobic conditions. Furthermore, to assess the interplay between phage and host, we examined changes in infection dynamics and one-step curves across aerobiosis and anaerobiosis

## MATERIALS AND METHODS

### Bacteria, phage, and growth conditions

*Salmonella enterica* subspecies *enterica* sv. Enteritidis s25pp strain (GenBank accession number MUNA00000000.1) was previously isolated by the Colombian Integrated Program for Antimicrobial Resistance Surveillance group, at the Colombian Corporation for Agricultural Research (Corporación Colombiana de Investigación Agropecuaria, AGROSAVIA). *Salmonella* s25pp was initially selected due to its rapid growth and absence of prophage induction by ultraviolet light, and subsequently used for phage interaction tests (24). The bacterial stock was conserved in 10% glycerol at −80°C. Bacteriophage ϕSan23 was previously isolated, characterized, and sequenced (31). Following the approach used by Kropinski et al. (32), phage suspension was maintained in buffer SM (5.8 g NaCl, 2 g MgSO_4_-7H_2_O, 50 mL Tris HCl 1 M pH 7.5, 5 mL gelatin 2%) at 4°C and its concentration was determined by serial dilution on double agar plaque assays (33).

### Growth conditions

*S*. Enteritidis s25pp was grown in liquid, semisolid, and solid media to perform different assays. For growth in the liquid medium, both aerobic and anaerobic experiments were performed in 20 mL tubes containing 10 mL of nutrient broth (NB) (Sharlau, Spain) at 37°C. In the anaerobic condition, the culture media were sparged with nitrogen, the tubes sealed with butyl rubber stoppers, and the headspace was filled with a mix of CO_2_ 20%: N_2_ 80%. In aerobic condition, culture media were not gassed.

Colony-forming units (CFU) and plaque-forming units (PFU) from aerobic and anaerobic cultures were quantified in nutrient agar plates by supplementing NB with 1.5% and 0.4% of bacteriology agar, respectively. The plates were incubated aerobically at 37°C. When quantifying bacteria or phages after plating samples from aerobic and anaerobic cultures, the incubation of plates was always conducted in aerobic conditions. This was done to determine the effect of anaerobiosis on the phage infection and the number of bacterial cells in the liquid culture assay previously described and avoid possible interferences of oxygen differences during the plating phase.

### Infection curve

One hundred microliters from a 1:10 dilution of overnight (ON) (16 h) bacterial cultures was used to inoculate 10 mL of fresh NB medium (Sharlau, Spain). After 3 h (absorbance 0.02 for anaerobic and 0.06 for aerobic cultures), the bacteriophage ϕSan23 was added to a multiplicity of infection (MOI) of 0.01. Progression of the infection was recorded every hour for 9 h, by measuring absorbance at 550 nm and calculating the CFU per milliliter against a reference growth curve (bacteria growing without phage) (33). The infection curve was carried out in aerobic and anaerobic conditions at 37°C, as previously described in growth conditions. Having sampled every time point, both sets of samples were plated and incubated in aerobiosis for 18 h at 37°C. The slope in the infection curve between hours 4 and 6 was calculated using linear regression in R software.

### One-step growth assay

The one-step assay was carried out according to Hyman and Abedon (33) under anaerobic and aerobic conditions using an MOI of 0.01. Samples for the viral count were taken every 5 min for 50 min to obtain the burst size, eclipse period, and latent period. To determine the eclipse period, chloroform was added to a replica set of samples, following Hyman and Abedon (33).

### Cell size measurement

Ten milliliters of fresh NB medium was inoculated with 100 µL of a 1:10 ON dilution. Bacteria were cultivated for 6 h and then the differences in cell size between aerobic and anaerobic growth were established by fluorescence microscopy. The cells were marked with DAPI (4′,6-diamidino-2-phenylindole): 5 mL from ON were centrifuged at 13,000 rpm, 4°C for 1 min. The supernatant was removed and 100 µL of 3 µM DAPI was added. The pellet was resuspended and incubated for 10 min at room temperature. The samples were washed twice with 200 µL PBS (phosphate-buffered saline) (13,000 rpm, 4°C for 1 min). The software Fiji-ImageJ 1.51u was used to calculate cell size. An additional measure was obtained by flow cytometry in Accuri C6 Flow Cytometer (Becton Dickinson). Cells were stained with DAPI as described, and characterized by their relative size (forward scatter, FSC) and relative granularity (side scatter, SCC).

### Plaque morphology

The effect of the absence of oxygen on plaque morphology was evaluated. A stock solution of ϕSan23 at 1 × 10^10^ PFU per milliliter was diluted six times in 10-fold serial dilutions (1:10). Dilutions 10^−3^ to 10^−6^ were plated mixing 100 µL of an ON culture and 100 µL of each phage dilution in 4 mL of soft agar. The plaques were observed after 24 h of incubation at 37°C in aerobic and anaerobic conditions. The plates in anaerobiosis were incubated in a Coy chamber (COY Laboratory Products) with an atmosphere of 99.7% N_2_.

### Resistance development

We evaluated the frequency of the appearance of resistant bacterial variants under aerobic and anaerobic conditions. *Salmonella* s25pp was infected (MOI 0.01) with ϕSan23 bacteriophage after 3 h of growing in NB medium. Four and 24 h after infection, the samples were plated, and 20–50 colonies were recovered from each of the five replicates to finally obtain 300 colonies from each oxygen condition. Resistance to the wild-type bacteriophage was tested by the small drop (or spot) assay, using a drop of 10 µL of ϕSan23 phage solution at a concentration of ~1 × 10^10^ PFU per milliliter (33). To calculate the ratio for each oxygen condition, the number of resistant isolates was divided by the total number of isolates evaluated. An isolate was considered resistant if the spot did not produce lysis.

### Western blot—FstZ and BtuB detection

Fresh NB medium was inoculated as previously described for the cell size measurement experiment. At a cell concentration of 5 × 10^7^ CFU per milliliter, after 6 h of growth with absorbances of 0.18 for the anaerobic and 0.68 for the aerobic experiment, 10 mL from both the aerobic and anaerobic cultures was centrifuged at 8,500 rpm, 4°C for 30 min. The pellet was resuspended in 500 µL of PBS and sonicated by 30 cycles of 10 s at 245 µm. Before cycles 1, 15, and 30, 5 µL of proteases inhibitor phenylmethylsulfonyl fluoride 1 mM was added. Next, the suspensions were centrifuged at 14,000 rpm for 15 min, and 400 µL of supernatant was transferred to clean tubes. Protein concentration was measured by the Bradford assay (Bio-Rad Protein Assay, Bio-Rad Laboratories, Inc., USA). Total protein suspensions were adjusted to a final concentration of 150 µg/mL for the following detection assays.

Western blot assays were developed to detect FtsZ and BtuB proteins. Each total protein suspension (protein concentration of 150 µg/mL) was mixed with 4× SDS sample buffer to obtain a final volume of 100 µL, and the mixture was boiled at 95°C for 10 min. To separate proteins, a 9% gel containing trichloroethanol 0.01% (vol/vol) was prepared, and 10 µL of the sample was added to each well and run at 90V for 10 min and 140V for 55 min, as described by Zeitler et al. (34). Total proteins were visualized using the ChemiDoc MP ImageLab software (Bio-Rad Laboratories, Inc., USA) (34). Following visualization, the proteins were transferred to PVDF (polyvinylidene fluoride) membranes to detect FtsZ and BtuB proteins. Rabbit anti-FtsZ (CUSABIO, USA) in a dilution of 1:10,000 and rabbit anti-BtuB (CUSABIO, USA) in a dilution of 1:3,000 were used as primary antibodies. A goat anti-rabbit horseradish peroxidase conjugate in a dilution of 1:10,000 was used as the secondary antibody (CUSABIO, USA). Next, the Immobilon Western Chemiluminescent HRP Substrate (Millipore, USA) for antibody detection and the ChemiDoc MP Imaging System (Bio-Rad Laboratories, Inc., USA) for stain-free activation and signal detection were used. Finally, the semi-quantitative analysis protocol designed by Zeitler et al. was applied to estimate the amount of FtsZ and BtuB proteins in aerobic and anaerobic conditions (34).

### DNA extraction and *btuB* gene sequencing

Thirty-six bacterial isolates from the aerobic cultures (17 phage-sensitive and 19 phage-resistant) and 27 isolates from the anaerobic cultures (19 phage-sensitive and eight phage-resistant) were selected. DNA was extracted from four colonies of each isolate, placed in 100 µL of sterile distilled water, and brought to a heating step at 95°C for 15 min followed by a cooling step on ice for 10 min and centrifugation at 13,000 rpm for 2 min. Fifty microliters of the supernatant containing the DNA was collected. The *btuB* gene was amplified as follows: 0.1 µL of 5 U/µL Platinum Taq DNA Polymerase High Fidelity (Invitrogen, USA), 2.5 µL of 10× High Fidelity PCR buffer, 1.0 µL of 50 mM MgSO4, 0.5 µL of 10 mM dNTPs Mix, 0.5 µL of 10 µM of each primer (btuB_f 5´- TATTGATTGACGGCGTGCGT-3´ and btuB_r 5´- AGCTGCCAGACAAGGTGTAT-3´), 1.0 µL of DNA solution, and sterile water to make up a volume of 25 µL. The PCR was carried out in a C1000TM Thermo Cycler (Bio-rad Laboratories, Inc., USA) as follows: initial denaturation at 95°C for 3 min, 34 cycles of denaturation at 95°C for 45 s, annealing at 60°C for 45 s and extension at 72°C for 60 s, and a final extension at 72°C for 7 min. The resulting PCR products were analyzed in a 1.0% agarose gel electrophoresis to determine their integrity and sequenced using Sanger technology in an ABI PRISM 3500 XL sequencer (Applied Biosystems, USA) at the Gencore (Universidad de los Andes, Bogotá, Colombia) sequencing center. The quality of the sequences was verified using Chromas v2.6.6 and confirmed as *btuB* gene sequences by a similarity search using blastn v2.6.0 (identify >99%) (35).

### Analyses of SNVs

To easily evaluate the quality of the generated sequences, ab1 files were converted to fastq using EMBOSS Seqret v6.6.0 (36). The quality of the resulting fastq files was analyzed using Fastqc v0.11.9 (37). Trimmomatic software v0.39 (38) was used to remove low-quality bases at the beginning and end of the sequences (CROP:810 HEADCROP:10). For each sample, R1 and R2 reads were assembled by CAP3 v10.2011 (39) and full-length sequences representing the entire amplicon size were obtained (1856 bp). Sequences were then aligned using Muscle v3.8.15 (40) and the corresponding alignment was used as input for finding single-nucleotide variations (SNVs), for which SNP-sites v2.5.1 was implemented (41). Finally, Tassel software v5.0 (42) was used to visualize the regions of variation among the sequences.

### *btuB* gene RT-qPCR

For RNA extraction, NB was inoculated with 100 µL of a 1:10 ON dilution of *Salmonella* s25pp. After 6 h of growth, 1.5 mL of the aerobic culture and 3 mL of the anaerobic culture were centrifuged at 13,000 rpm, 4°C for 2 min. The pellet was resuspended in 750 µL of Trizol Reagent and the RNA extractions were developed following the manufacturer’s instructions. To verify the RNA integrity, an aliquot of the sample was run on agarose gel (1.2% agarose, 100V 5 min, and then 80V for 15 min). Total RNA extracts were treated with DNase, and the RNA concentration was calculated using NanoDrop 2000 (ThermoFisher, USA). All the samples were normalized to 45.7 ng/µL and the cDNA was synthetized using the RevertAid First Strand cDNA Synthesis kit (ThermoFisher, USA).

The 16S rRNA gene was used as a housekeeping gene according to Wang et al. to obtain an amplicon of 123 bp (forward: GTTACCCGCAGAAGAAGCAC, reverse: CACATCCGACTTGACAGACC) (43). To detect the differential expression of the *btuB* gene, primers were designed to obtain a 119 bp amplicon (forward: TACCGATACGCCATTACCGC, reverse: CCGAATCATAGCGGGAACCA). SYBR Green was used to detect the amplification in real-time as follows: initial denaturation at 94°C for 3 min, 39 cycles of 94°C 10 s, 60°C 30 s. Calibration curves for each primer and melting curves between 44°C and 95°C were developed. Data were analyzed by applying the Pfaffl method (44). First, the difference in C_T_ values between experimental and control samples for *btuB* and 16r rRNA genes was calculated, along with the amplification efficiency for each primer set. Next, we calculated the fold change in gene expression between experimental and control samples for the target and housekeeping genes, to finally normalize the fold changes. The relative expression of the *btuB* gene was analyzed in triplicate.

### RNA-seq

RNA sequencing was performed to obtain the transcriptome of the bacteriophage and bacteria during viral life cycle in aerobic and anaerobic conditions. Tubes with 10 mL of fresh NB medium were inoculated with 100 µL of a 1:10 ON dilution. Following 3 h of incubation (absorbance 0.02 for anaerobic and 0.06 for aerobic cultures) at 37°C and 100 rpm, the bacteriophage ϕSan23 was added to an MOI of 1. According to the results of the one-step curve, for aerobic infection at 0 min (pre-infection), 5, 10, and 15 min post-infection the tubes were immersed in liquid nitrogen to stop the metabolism in the bacteria. For anaerobic infection, one more sample was taken 20 min post-infection. Then, using the frozen samples, total RNA extraction was performed with the SV Total RNA Isolation System kit (Promega, USA). Each sample was an independent tube in triplicate per time. The quality control of RNA extraction was verified in agarose gel and NanoDrop 2000 (ThermoFisher, USA).

The library preparation, rRNA depletion, and sequencing were performed at the Genome Technology Access Center, Washington University, using RNA-seq library preparation with Qiagen FastSelect 5S/16S/23S rRNA depletion. Paired-end sequencing (2 × 150) was performed on Illumina NovaSeq.

The quality of raw reads was evaluated using fastqc v0.11.9. Subsequently, adapters and low-quality reads were removed by Trimmomatic v0.39 (SLIDINGWINDOW:4:30, MINLEN:100, HEADCROP:10) (38). Reference genomes of ϕSan23 (31) and s25pp (GCA_002105275.1 RefSeq) were re-annotated using Rastk v2.0 (45) and eggNOG mapper v2.1.5 (46), respectively. For EggNOG mapper, mmseqs (47) was used on blastx mode for gene prediction and annotation (--genepred search, -m mmseqs), whereas Rastk was run on default mode. Reference genomes and their annotations were used as templates to map the trimmed sequences using Star v2.7.9a (48). HTSeq v0.6.1p1 (49) was used to extract the number of reads mapping to the coding sequences (-s no -r pos -t CDS -i ID). Further table formatting and plotting were made on R using the Tidyverse suite.

### Statistical analysis

Statistical significance for cell size and protein detection differences between aerobic and anaerobic conditions were determined by the *t*-test. The level of statistical significance was *P* < 0.05. Differential expression analysis was carried out using Deseq2 v1.28.1 (50).

## RESULTS

### Bacterial growth and cell size in aerobic and anaerobic conditions

**Fig 1:**
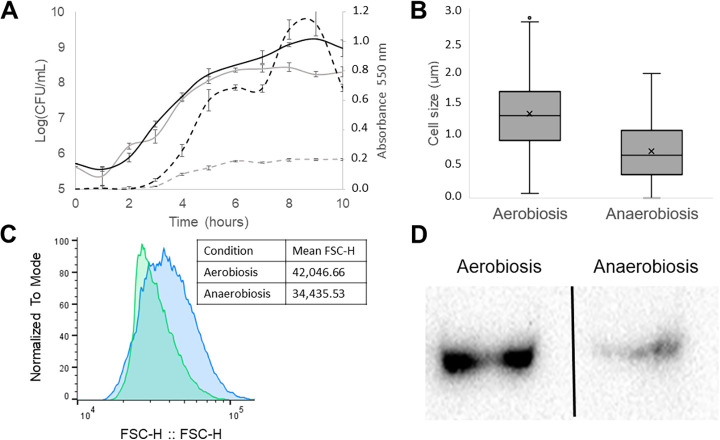
Effect of anaerobiosis in bacterial growth and cell physiology. (**A**) Growth curve of *Salmonella* s25pp. Continuous line: biomass in log (CFU per milliliter), discontinuous line: absorbance to 550 nm. Black lines: aerobic, gray lines: anaerobic. (**B**) Cell size distribution of *Salmonella* s25pp growing in nutrient broth medium in aerobic and anaerobic conditions. X represents the mean in each data set. (**C**) Flow cytometry. Histogram of FSC-H values of cells in liquid medium (NB) after 6 h of growing at 37°C. Blue: aerobic condition, green: anaerobic conditions. (**D**) Western blot assay of FtsZ protein expression in *Salmonella* s25pp. Left: aerobic conditions, right: anaerobic condition. Each experiment was performed in triplicate.

Initially, we assessed the growth kinetics of *Salmonella* s25pp in aerobiosis and anaerobiosis (Fig. 1A). The absorbance readings indicated that the growth rate was higher in the presence of oxygen. However, the CFU counts did not show any significant difference during the first 6 h. After this time point, bacteria continued to grow at a lower rate in aerobic conditions, while it reached the stationary phase in anaerobiosis.

The surprising discrepancy between absorbance and CFU counts led us to explore whether this could be explained by differences in cell size. We dyed the bacteria with DAPI and calculated the 2D area of approximately 220 bacterial cells from each oxygen condition. According to the measurements, the length of bacteria growing in presence of oxygen is 1.352 + 0.579 µm while in its absence is 0.750 + 0.472 µm (Fig. 1B), with bacterial cells in anaerobiosis being significantly smaller (t-Student *P* < 0.05). To verify these results, flow cytometry analysis was performed. The histogram of FSC-H values (Fig. 1C) shows that size distribution in aerobic growth is wider, possibly associated with a non-homogeneous distribution of oxygen inside the tubes. The average FSC-H value, which is related to the size, is larger in aerobiosis than it is in anaerobiosis (mean value of FSC-H in aerobiosis is 42,046.66 and in anaerobiosis 34,435.53), confirming that *Salmonella* s25pp cells are smaller when grown in the absence of oxygen.

Several authors have suggested that the amount of FtsZ proteins, part of the Z ring and essential in cell division, correlates with cell size (29, 30). Thus, we evaluated the amount of FtsZ protein in *Salmonella* s25pp when grown in the presence/absence of oxygen. The Western blot assay (Fig. 1D; Table 1) shows a difference in the amount of FtsZ protein in both conditions, with bacteria in anaerobic conditions exhibiting a reduced amount of the protein. In the semi-quantitative analysis, the immunoblot was normalized with the protein gel (Fig. S1; Table S1 supplementary materials) and relative quantitation of FtsZ protein was obtained to compare the expression of the protein in different oxygen conditions. This analysis showed that in anaerobiosis, *Salmonella* s25pp shows a reduction of 56.36% (*P* < 0.05), on average, in the expression of the FtsZ protein. Overall, the data consistently showed the effect of anaerobiosis on *Salmonella* s25pp cell size.

**TABLE 1:** Semi-quantitative analysis in Western blot assay (34) of FtsZ protein expression in *Salmonella* s25pp^*a*^

Anaerobic replicates	Normalization factor	Relative quantification in relation to each aerobic replicate			Percentage of FtsZ expression in anaerobiosis (compared to aerobiosis)
		R1	R2	R3	
R1	0.6708	1.4757	0.5557	0.6153	68.60%
R2	0.7141	0.6758	0.2545	0.2818	33.44%
R3	0.7389	0.5638	0.2123	0.2351	28.87%
				Average	43.64%

^*a*^ Each replicate represents an independent protein extraction. Each experiment was performed in triplicate.

### Phage infection

**Fig 2:**
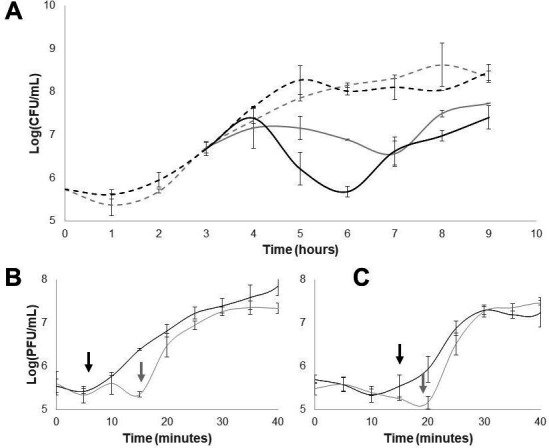
(**A**) Infection curve of phage ϕSan23 in *Salmonella* s25pp. The phage was added after 3 h of bacteria growth. Continuous lines: bacteria with phages. Discontinuous lines: bacterial growth without phages. Black lines: aerobic; gray lines: anaerobic. (**B and C**) One-step curves of phage ϕSan23 under aerobic and anaerobic conditions. Black lines: aerobic, gray lines: anaerobic. (**B**) Plating with chloroform, the arrows indicate the eclipse periods. (**C**) Plating without chloroform, the arrows indicate the latent periods. Each experiment was performed in triplicate.

Infection curves and one-step assays were performed to analyze the effect of oxygen in the infection and viral replication cycle of ϕSan23. The infection curve (Fig. 2A) showed that the reduction of bacterial population per time unit (RBPT) in aerobiosis occurs faster: the bacterial population is reduced one logarithmic unit more than it is under anaerobic conditions, and this reduction is achieved 1 h earlier. The bacterial reduction slope in the infection curve is steeper in aerobiosis between hours 4 and 6 (-0.85 in aerobiosis and −0.14 in anaerobiosis). Thus, oxygen in the environment furthers the infection. Nevertheless, resistant variants appeared in both cases, as the population of *Salmonella* increased after 3–4 h of infection (following the addition of the phage) (Fig. 2A). One-step curves showed that the lack of oxygen affects the replication of ϕSan23 since the viral progeny is reduced, and the eclipse and latent period are longer: the eclipse period lasts 5 min and the latent period 15 min, while the burst size is 260. In contrast, in anaerobiosis, the eclipse period lasts 15 min, the latent period 20 min, and the burst size is reduced to 114 (Fig. 2B and C). Furthermore, the plaques with no oxygen were larger than aerobic plaques, with a clear center but a fuzzy and cloudy border (Fig. S2).

### BtuB receptor

**Fig 3:**
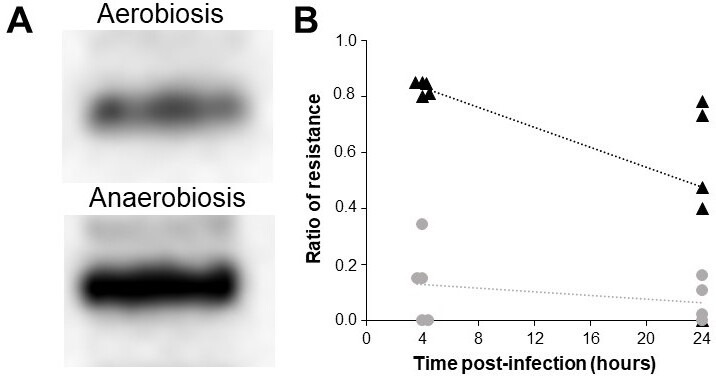
(**A**) Western blot. Expression of BtuB protein in *Salmonella* s25pp. (**B**) Proportion of resistant bacteria clones to the phage ϕSan23 after 4 h and 24 h post-infection in liquid medium. Black triangles: aerobic condition, gray circles: anaerobic condition. Each experiment was performed in triplicate.

Since the infection curve showed that the RBPT is affected in anaerobiosis, we hypothesize that the absence of oxygen might cause a BtuB protein (phage receptor) reduction on the cell surface and this reduction will contribute to the observed differences. To test this, we evaluated whether differences at the transcription or translation level could be detected for the *btuB* gene. The RT-qPCR showed that in anaerobiosis, the *btuB* gene is expressed 1.7-fold more than in aerobiosis (Table 2). Moreover, a semi-quantitative analysis of the Western blot showed that the expression of BtuB protein in anaerobiosis is approximately 87% higher on average (*P* < 0.05) (Fig. 3A; Table 3) (normalized using the protein gel, Fig. S3; Table S2 supplementary materials). Thus, the *btuB* gene is upregulated in anaerobiosis and the protein also is overexpressed. These results suggest that the lower RBPT in anaerobic conditions is caused by factors other than the availability of receptors present on the cell surface.

**TABLE 2:** RT-qPCR Ct for housekeeping 16S rRNA and *btuB* gene and *btuB* fold change in *Salmonella* s25pp in anaerobiosis compared with aerobiosis, according to Pfaffl method^*a*^

Aerobiosis		Anaerobiosis		*btuB* fold change anaerobiosis:aerobiosis
16S (Ct)	*btuB* (Ct)	16S (Ct)	*btuB* (Ct)	1.7
17.61	32.98	21.36	35.88	

^*a*^ Each experiment was performed in triplicate.

**TABLE 3:** Semi-quantitative analysis in Western blot assay (34) of BtuB protein expression in *Salmonella* s25pp^*a*^

Anaerobiosis replicates	Normalization factor	Relative quantification in relation to each aerobic replicate			Percentage of protein expression
		R1	R2	R3	
R1	1.3232	0.8568	0.6735	0.9783	102.99%
R2	1.3125	1.3912	1.0936	1.5885	165.87%
R3	1.3135	2.4581	1.9322	2.8068	293.31%
				Average	187.39%

^*a*^ Each replicate represents an independent protein extraction. Each experiment was performed in triplicate.

### Resistance development

As shown by the infection curve (Fig. 2A), resistant clones of bacteria appeared some hours after infection in both oxygen conditions, leading to an evaluation of the development of resistance at 4 h and 24 h after phage infection. We chose the 4-h time point after infection for clone isolation, that it aligns with 7 h of bacterial growth in Fig. 2A, a period during which the most significant variations in bacterial population reduction under the two oxygen conditions were noted. For resistance testing, colonies were chosen from each time point and condition. Specifically, at 7 h of bacterial growth, 152 colonies were selected from the aerobic conditions and 160 colonies from the anaerobic conditions. At 24 h of bacterial growth, 157 colonies were chosen from the aerobic condition assays, and 146 colonies from the anaerobic ones.

The results indicated that the proportion of resistant colonies in anaerobic conditions was consistently lower and remained relatively stable over the assessed period of time. Moreover, the resistance ratio (Fig. 3B) shows that there were no important differences between replicates in anaerobiosis 24 h post-infection. However, at 24 h post- infection in aerobic conditions, one replicate is totally sensitive to the phage and the others have a great proportion of resistant clones. Meanwhile, in the case of aerobiosis, all the replicates are almost resistant to the phage 4 h after infection. This supports the behavior of the infection curve where the bacterial population increased at this time point.

We evaluated whether changes in the ϕSan23 phage receptor (BtuB) (24) were involved in the resistance. To this end, we sequenced the *btuB* gene of the bacterial variants recovered. The analyses showed that the *btuB* gene had no associated SNVs at any of the time points (4 h and 24 h post-infection) with the oxygen condition nor with phage resistance (Fig. S4 and S5). Therefore, the bacterial resistance is mediated by mechanism(s) other than mutations in the phage receptor in both aerobiosis and anaerobiosis.

### RNA-seq

RNA sequencing allowed us to obtain the transcriptome of *Salmonella* s25pp and phage ϕSan23 during one infection cycle (data set is available in the supplementary material). The transcriptome was analyzed for the aerobic and the anaerobic infection, and also at relevant time points according to the one-step curve results, considering that the viral life cycle in anaerobiosis is longer. Thus, for the aerobic infection, 0 (pre-infection), 5, and 15 min post-infection time points were evaluated, while for the anaerobic infection, 0 (pre-infection), 5, 15, and 20 min post-infection time points were evaluated. Additionally, the bacterial transcriptomes without phage infection were compared to analyze the effect of oxygen on the bacterial metabolism.

#### The effect of oxygen on the bacterial growth

The transcripts of *Salmonella* s25pp in anaerobic growth without infection showed that 195 genes were upregulated (log2foldchange > 2) while 273 were downregulated (log2foldchange < −2) in comparison to the aerobic condition. The genes were annotated and classified in COG (Clusters of Orthologous Groups) categories (Fig. S6A) The COG category with the most differentially expressed genes was, as expected, energy production and conversion. In the energy category, genes to ferredoxin proteins such as *hycBDEFGO, hydN, hydAB, asrA*, *pshB,* and fumarate reductase genes (*frdADC*) were upregulated. In agreement with the overexpression of these genes, the expression pattern of other genes indicated that bacteria use formate as the electron donor and thiosulfate and dimethyl sulfoxide as electron acceptors in anaerobic growth. Consistently, genes of the tricarboxylic (TCA) cycle (*sucABD, icd, mdh, acnA, fumAC*) and oxidative phosphorylation (*cyoABC, sdhABCD, cybC*) for aerobic respiration were downregulated.

The infection causes changes in the bacterial metabolism both in aerobic and anaerobic conditions. In the first 15 min of anaerobic infection, 190 genes were upregulated and 157 genes were downregulated compared to aerobic infection, although the general profile of differential expression did not reveal any drastic changes (Fig. S6B).

#### Aerobic infection

**Fig 4:**
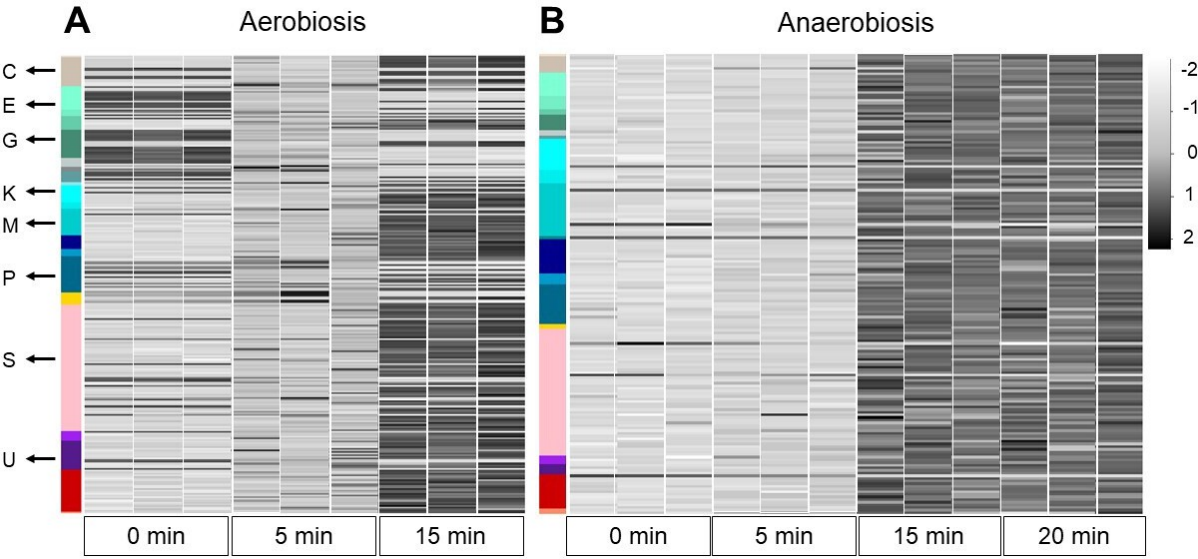
Differentially expressed bacterial genes (−2.5 > fold change, 2.5 < fold change, *P*-value adjustment <0.05) during phage infection. (**A**) Aerobic infection. (**B**) Anaerobic infection. In descending order by COG categories. A: RNA processing and modification, C: energy production and conversion, E: amino acid transport and metabolism, CE/EGP/ET: genes grouped in more than one category, F: nucleotide transport and metabolism, G: carbohydrate transport and metabolism, H: coenzyme transport and metabolism, HI/HP/HQ: genes grouped in more than one category, I: lipid transport and metabolism, IQ: genes grouped in more than one category, J: translation, ribosomal structure, and biogenesis, K: transcription, L: replication, recombination, and repair, M: cell wall/membrane/envelope biogenesis, NPTU/NU: genes grouped in more than one category, O: post-translational modification, protein turnover, chaperones, P: inorganic ion transport and metabolism, Q: secondary metabolite biosynthesis, transport, and catabolism, S: function unknow, T: signal transduction mechanism, U: intracellular trafficking, secretion, and vesicular transport, Unknow: without COG category, V: defense mechanism. Black: upregulated, white: downregulated. The proportion of upregulated genes in the anaerobic infection (308 upregulated/26 downregulated) was greater than in the aerobic infection (286 upregulated/124 downregulated). Nonetheless, the total number of genes differentially expressed was lower in the anaerobic infection.

In the case of aerobic infection, the category with the highest number of differentially expressed genes was the COG unknown function category. (Fig. 4A). Surprisingly, genes related with nitrate reduction (nitrate and nitrite reductases needed for anaerobic respiration) were upregulated in aerobic infection (Fig. S7). Furthermore, the anaerobic facultative regulon *arcA* was also upregulated in the aerobic infection. Other genes upregulated in the aerobic infection were *osmB,* a lipoprotein involved in osmotic stress (51), and *cydA*, a terminal oxidase that produces a proton motive force in the inner membrane when cells are grown at low aeration (52). Interestingly, this oxidase is less sensitive than other cytochrome oxidases to nitric oxide and reactive oxygen species (53).

**Fig 5:**
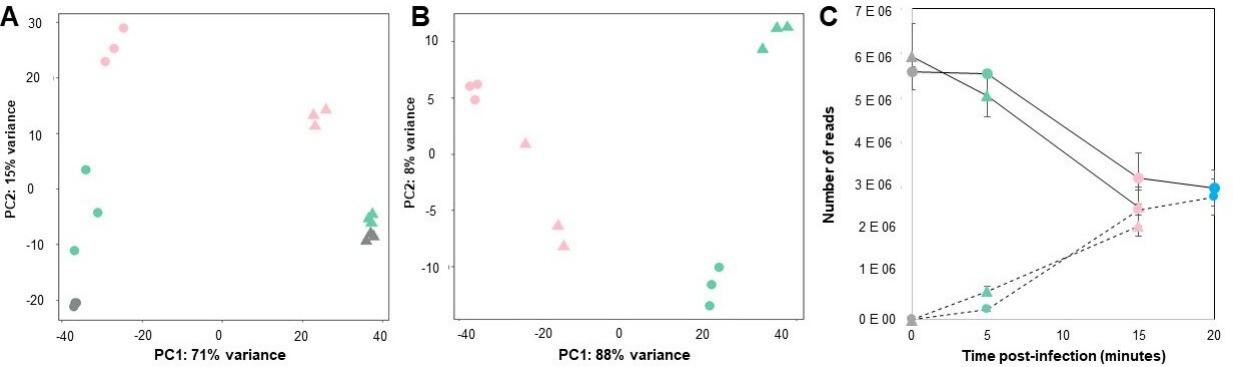
Principal components plot. (**A**) Bacterial transcripts during infection, (**B**) phage transcripts during infection. (**C**) Number of reads (transcripts) of bacterium and phage per oxygen condition over time. Continuous line: bacterial transcripts, discontinuous line: phage transcripts. Circles: aerobic condition, triangles: anaerobic condition. Gray: 0 min, green: 5 min, pink: 15 min, blue: 20 min post-infection.

Among the downregulated genes in the aerobic infection, we found *cstA*, a gene related to the use of peptides as a source of carbon (54). Additionally, the succinate dehydrogenase complex *sdhADC* needed for aerobic respiration was downregulated during aerobic infection.

#### Anaerobic infection

As the aerobic infection, the anaerobic infection showed that some genes were expressed gradually over the time (Fig. 4B and 5A). However, few differences were observed between 5 min and 10 min, and between 15 min and 20 min. When comparing 5 and 15 min, changes in expression occurred but these were not associated with a particular COG category. Once again, the unknown function category covered the biggest representation of genes, but fewer genes in energy production and conversion were reported as differentially expressed. Interestingly, only two genes associated with nitrate reduction (*narW, narI*) were upregulated. Genes *cysP* and *cysT*, involved in the transport of thiosulfate and sulfate, respectively, were also upregulated. The expression of these transporter genes coincides with the expected type of anaerobic metabolism that *Salmonella* s25pp uses during its anaerobic growth.

The genes *astA, astB, astD*, and *astE* of the arginine succinyl transferase (AST) pathway were upregulated only in anaerobic infection, while in the anaerobic culture without phage infection, these genes were actually downregulated. The AST pathway is an arginine catabolic route to use amino acids as a sole nitrogen source. Additionally, genes *argD, argF,* and *argO* associated with biosynthesis and arginine transport were also overexpressed only in anaerobic infection, as well as the potassium transport system *kdpA-kdpB*. Finally, the gen *cstA* was downregulated as in the aerobic infection.

Independently of the oxygen availability, the putative genes *rtcA* and *rtcB* were overexpressed during phage infection. *rtcA* and *rtcB* encode the RNA 3´-phosphate cyclase and RNA ligase, respectively (55, 56). Counterintuitively, genes for restriction-modification, CRISPR-Cas, modification-based systems, and other genes like *lexA* or *recA* related with microbial antiviral defenses were downregulated or not differentially expressed during the infection. Nonetheless, the toxin/antitoxin system *dinJ-yafQ* was overexpressed in the anaerobic infection, a toxin/antitoxin system type II (57). The antitoxin gene *dinJ* was upregulated in both aerobic and anaerobic infections, but the toxin *yafQ* was only upregulated in the anaerobic one.

#### Phage transcriptome

Phage genes are expressed differentially in time (Fig. 5B; Fig. S8 and S9). In both oxygen conditions, in the last minutes of viral cycle, structural (COG category X), amino acid transport and metabolism, and *tRNA* genes are overexpressed, while in the early minutes, genes for nucleotide transport and metabolism are strongly expressed. Many unidentified genes (classified as phage proteins) also showed high levels of expression in the different phases of phage infection. The expression profile of phage genes for aerobiosis and anaerobiosis was very similar but required a longer time in anaerobiosis (Fig. S8 and S9) A differential analysis was performed for 15 min in the aerobic infection and 20 min in anaerobic infection, but no differences were found. In aerobiosis, 56 genes were differentially expressed (40 upregulated and 16 downregulated) and in anaerobiosis, 82 genes were differentially expressed (56 upregulated and 26 downregulated). Unfortunately, most of these genes are not annotated and their functions remain unknown. To enhance phage protein annotation, a strategy centered around hidden Markov models (HMMs) was employed using hmmscan v3.3.2, with the PHROGs database as the reference point. In essence, this database comprises a compilation of HMM models derived from approximately 38,000 orthologous protein groups found in phages and archaeal viruses (58). Regrettably, these endeavors yielded no discernible improvements in the annotation process.

## DISCUSSION

*Salmonella* is one of the major causes of gastroenteritis transmitted by food in humans (59, 60). Infections by *Salmonella* sp. are usually associated with the consumption of poultry products, thus controlling this pathogen at different stages of the poultry production line is important in preventing gastrointestinal diseases. A promising alternative to control *Salmonella* is phage therapy using, for example, ϕSan23, as shown in previous farm tests (61), but one important aspect which is usually not considered is the fact that *Salmonella* is an enteric facultative bacterium inhabiting the animals’ intestines, where oxygen availability is limited. *Salmonella* can use electron acceptors other than oxygen while colonizing the intestine (17, 62, 63); however, an anaerobic metabolism represents less energy and possibly imposes metabolic stress on the bacteria. The effect of these changes on the phage infection or life cycle was not previously assessed. Our group published a review about phages in anaerobic systems (64) some years ago; in this review, we briefly mentioned our preliminary results regarding differences in cell size, plaque morphology, resistance development, burst size, and infection efficiency in a *Salmonella* phage. Here, we show the complete, confirmed results and their analyses.

Our experiments revealed changes in the physiology of *Salmonella* in the absence of oxygen. Bacteria cultivated without oxygen are smaller and the amount of the FtsZ protein is positively correlated with this reduced cell size. The association between cell size and the amount of FtsZ protein was described previously in *Escherichia coli* (29, 30), where it was demonstrated that the anoxic environment affects cell size as do temperature, growth rate, and the availability of nutrients (27, 28, 65). The discrepancies between absorbance and CFU counting (Fig. 1A) were also observed in other authors’ research: Loui et al. mentioned how absorbance at 550 nm of *arcA* and *arcB* mutants during the log phase was lower than it was in wild-type *E. coli* but the CFU counting was similar in mutants and wild type (although experimental data were not shown in the published paper); this lower absorbance was obtained both in aerobic and anaerobic growth conditions (66). Lu et al. (also, as data not shown in the paper) reported the same phenomenon in *arcA* mutant and wild type of *Salmonella enterica* serovar Enteritidis when the bacteria were cultivated in minimal and Luria-Bertani (LB) medium in aerobic and anaerobic conditions (67). Although these authors did not evaluate the association between *arcA*, *ftsZ*, and cell size, ArcA is one of the global regulation systems for anaerobic metabolism in anaerobic facultative bacteria and can indirectly regulate other pathways. Thus, the ArcA system could regulate the amount of FtsZ protein inside the cell and therefore indirectly control cell size in anaerobiosis. In the transcriptomic analysis, we found that *arcA* is upregulated (with a marginal fold2change of 1.76) and *ftsZ* gene is not expressed differentially in *Salmonella* s25pp under anaerobic conditions. Cell size depends on the accumulation of FtsZ protein and not only on its transcription (27), so other regulatory mechanisms have to be involved in higher FtsZ accumulation in anaerobiosis. It was also observed that the growth kinetic of *Salmonella* s25pp is similar in aerobiosis and anaerobiosis during the first 6 h. Lu et al. reported the same behavior in *Salmonella* when they were growing aerobically or anaerobically (data not shown in the published paper) (67). Thus, it can be concluded that smaller cell size does not affect the growth kinetics of *Salmonella* in anaerobiosis.

Given the strength of the phage-host relationship, metabolic or physiological changes in the bacteria must affect the phage infection process. Leisken et al. demonstrated that in *Yersinia enterocolitica*, temperature and MOI influence the infection (68). The current study showed that anaerobiosis also affects the interactions between phage and bacteria: infection and one-step curves presented differences when the infection occurs with or without oxygen. These results are also associated with changes in plaque morphology. Back in 1975, Mc Connell et al. (69) observed the same phenomenon in the plaques when *Salmonella* and *E. coli* were incubated in anaerobic jars. They proposed five possible reasons: changes in the latent period, changes in the burst size, changes in the growth rate, variation in the number of receptors on the cell surface, and a different diffusion coefficient of the phages in the agar. Results from our study indicate that the hypothesis of Mc Connell and collaborators were partially right: changes in the latent period and burst size were observed when the infection occurred in anaerobiosis. In contrast, growth curves showed that bacterial growth kinetics is similar in both oxygen conditions. The number of receptors on the cell surface increased in anaerobiosis; however, at present, we do not know how this relates to plaque morphology (Fig. 3A). A question yet to be answered and for which more information is needed considering all the mechanisms that regulate its expression and synthesis are associated with the theoretical amount of BtuB protein present on the cell surface. Regarding the possible changes in the agar diffusion coefficient, this, too, is yet to be evaluated. Another possible explanation could be the enhanced expression of lysins in anaerobiosis, which upon the lyse of the infected cells diffuses a greater distance and causes the lysis adjacent cells. In our assay of the ϕSan23 transcriptome during the infection in aerobiosis and anaerobiosis, we observed no differential gene expression in genes annotated as lysins.

We also demonstrated that the RBPT decreased in the anaerobic condition probably associated with a viral replication cycle that takes more time and a reduced viral progeny. Our initial hypothesis concerning the lower amount of BtuB receptor on the cell surface in anaerobiosis can be disregarded, meaning that the low RBPT is probably associated with a process that occurs after phage-receptor interaction. Still, some experimental findings presented here could explain why the RBPT decreased under limited oxygen conditions. On one hand, smaller cells could cause a lower probability of encounter between phage and bacterium. Eriksen et al. in a spatial model of phage-bacteria interaction in a semisolid medium showed how the size of the bacterial colonies affects the lysis in an environment with finite sources (70). Furthermore, Chong et al. demonstrated that elongated FtsZ-depleted *E. coli* DH5α cells absorb more T4 phages compared to shorter cells with functional FtsZ. On the other hand, if the number of phages released after each viral cycle is smaller compared to the aerobic condition, the reduction of the bacterial population will be less efficient over time. A fitting question here would be whether smaller cells are restricted in some ways to hosting larger amounts of viral progeny. Some studies have indicated that burst size can be influenced by the ratio of host cell volume to virus volume (71). In the case of phages, Choi et al. (72) observed that the burst size of T4 phage is greater in larger host cells, while Kannoly et al. (73) found a positive correlation between burst size and cell volume in lambda phage. In fact, the likelihood of lysogeny in lambda phage increases in small cells during both the stationary and exponential phases (74), possibly due to the larger viral progeny produced in larger cells. Therefore, the smaller burst size observed in smaller anaerobic cells in our results aligns with previous findings reported for *E. coli* phages.

**Fig 6:**
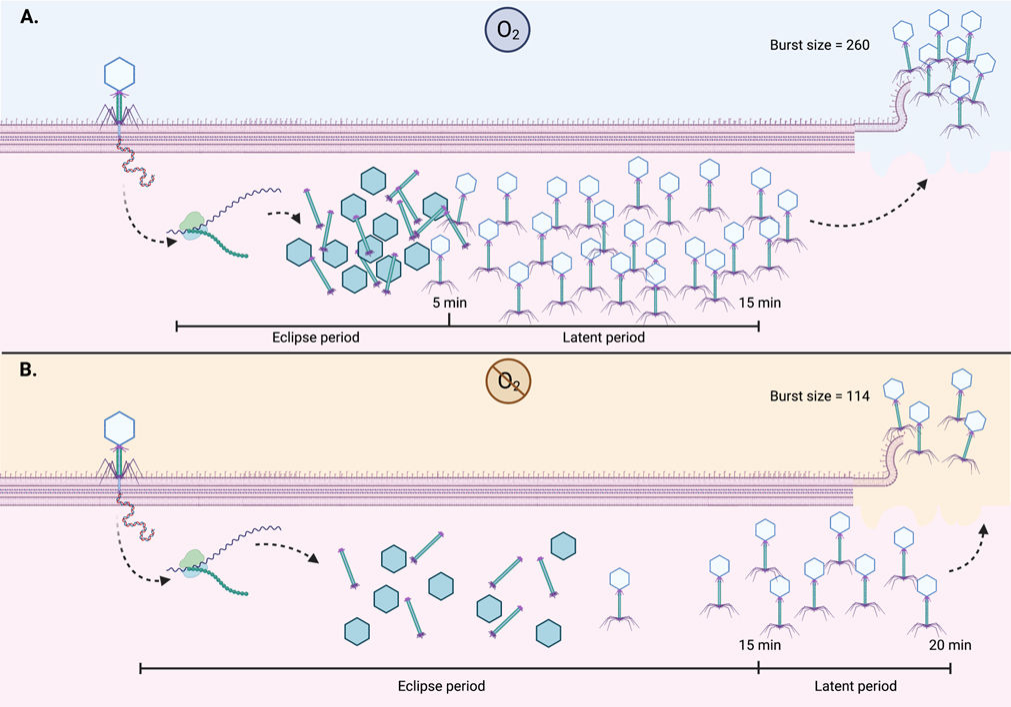
ϕSan23 replication cycle in *Salmonella* s25pp in aerobiosis and anaerobiosis. (A) Phage replication in aerobic conditions. (B) Phage replication in anaerobic conditions. The results show that the eclipse and latent period are longer in anaerobiosis, but the viral progeny is reduced.

One of the most interesting results obtained here was the modification of the viral replication cycle (Fig. 6); in anaerobiosis, the burst size is smaller although the eclipse and latent period are greater. Thus, phage replication is affected by the environmental parameters that the bacteria are subject to. The absence of oxygen in enteric facultative bacteria activates the FNR and ArcA systems that regulate anaerobic metabolism. In the case of *Salmonella* Typhimurium, these systems suppress pathways related to energy generation, amino acid, and fatty acid transport in aerobic metabolism, but activate gene expression of anaerobic metabolism, flagellar biosynthesis, motility, and sugar transport (15, 16). Therefore, in anaerobic environments, *Salmonella* undergoes many molecular and metabolic changes that might be challenging for phages. Indeed, our transcriptomic analysis confirms that, in anaerobiosis (without phage infection), many genes are expressed differentially (as we expected, the up/down expression ratio was lower than 1). However, in anaerobic phage infection, more genes were upregulated during first 15 min, compared to aerobic infection. During anaerobic growth, genes related to energy were differentially expressed but in phage infection, many differentially expressed genes were classified in the unknown function (S–COG) category. Thus, those metabolic changes caused by oxygen limitations during infection rely on genes whose metabolic function we do not yet understand.

**Fig 7:**
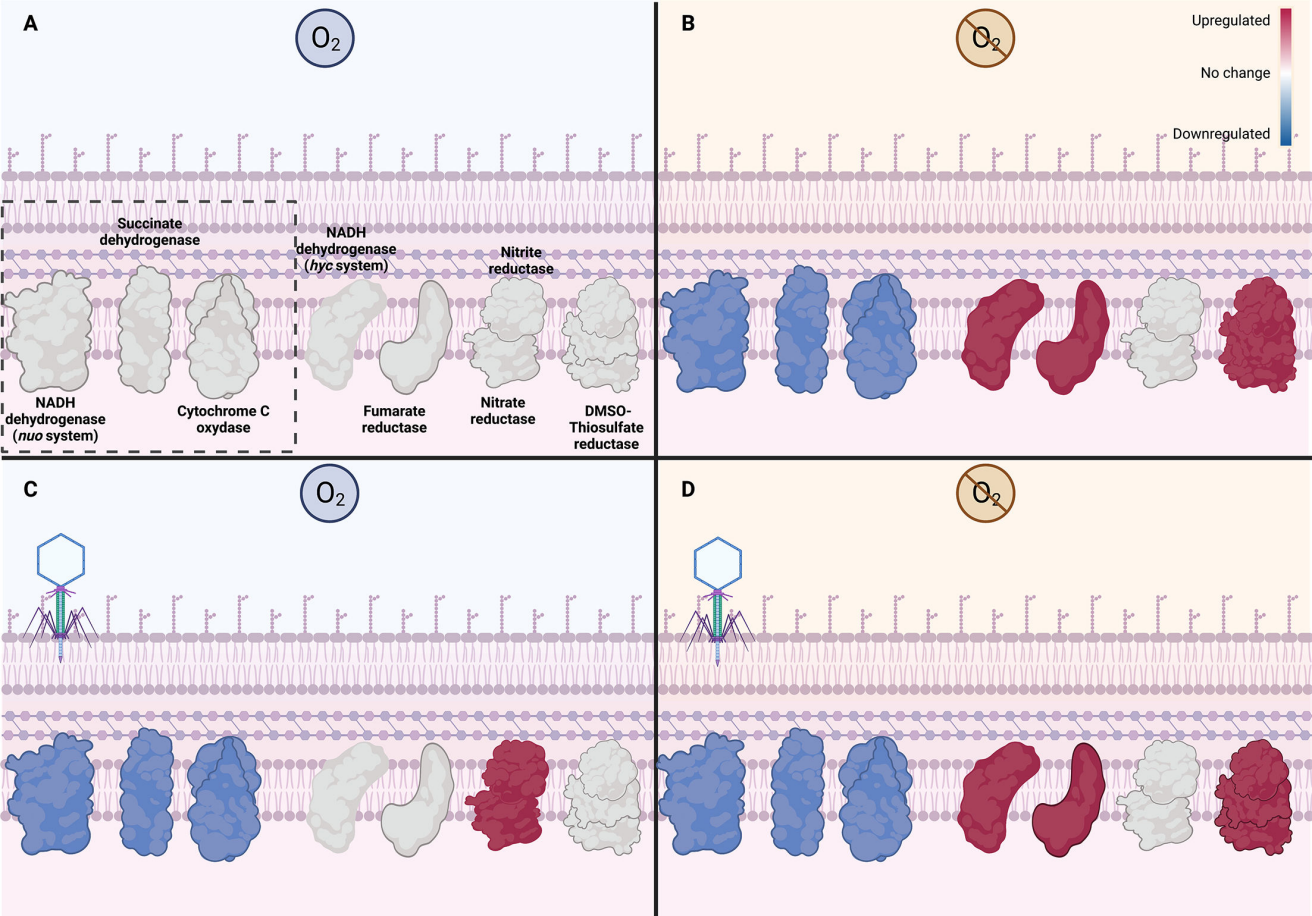
Changes in transcriptome of genes related to the energy metabolism in *Salmonella* s25pp induced by the absence of oxygen and/or the phage infection. Panel A is used as references for comparison of the enzyme expression in panels B, C, and D. Red: upregulated, blue: downregulated, white: no significant change in the expression or basal expression in the reference condition. (**A**) The reference expression corresponds to bacterial growth in aerobiosis without infection. The enzymes inside the square are expressed in aerobic growth mainly. (**B**) *Salmonella* s25pp grown in anaerobic conditions without infection. (**C**) *Salmonella* s25pp grown in aerobic conditions and infected with the phage ϕSan23. (**D**) *Salmonella* s25pp grown in anaerobic conditions and infected with the phage ϕSan23.

A shift to anaerobic metabolism leads to reduced energy production, which in turn could negatively affect viral replication. Indeed, previous studies show that genes related to energy generation are downregulated during phage infection in *Yersinia enterocolitica, Campylobacter jejuni*, and *Pseudomonas aeruginosa* (68, 75–77). But in *Clostridiodes difficile* (formerly, *Clostridium difficile*), a strictly anaerobic bacterium, energy pathways were upregulated under phage infection (78). Our transcriptomic results show that anaerobic respiration is induced in aerobic infection of *Salmonella* s25pp with the phage ϕSan23. Also, oxidative phosphorylation genes in aerobic respiration were downregulated (Fig. 7). Thus, energy production genes play an important role in phage infection, and each phage-host interaction associated with a specific environment defines how energy genes are controlled. Surprisingly, in this work in which we evaluated the effect of oxygen in phage infection, we found that ϕSan23 induced anaerobic metabolism in a facultative bacterium. This coincidence reveals that environmental conditions and phage strategies are correlated, and a potential co-evolution process has defined phage-host interactions.

Although using oxygen as a terminal electron acceptor is the more efficient way to produce energy, it has an oxidative stress cost caused by reactive oxidative species that can damage the DNA and have other negative effects on the cell. This oxidative stress may also affect viral replication during phage infection. For instance, phage ϕAbp1 infecting *Acinetobacter baumannii* induced the upregulation of stress genes involved in oxidation-reduction processes (79). In the case of the *C. jejuni* phage NCTC 12673, the infection in microaerobic conditions (85% N_2_, 10% CO_2_, 5% O_2_) is altered by oxidative stress. In their work, Sacher et al. observed that genes associated with removing reactive oxygen species (*katA*, *ahpC*, *sodB*) were upregulated during infection, and when these genes were mutated, plating efficiency decreased drastically (75). In *Salmonella* s25pp infection, transcriptomic analysis during infection in aerobiosis and anaerobiosis showed that the genes *katA*, *ahpC,* and *sodB* were not differentially expressed. Despite the expression of oxidative stress responses, genes did not change, the infection in aerobiosis meant that *Salmonella* s25pp used an anaerobic metabolism to produce energy. Additionally, during aerobic infection, succinate dehydrogenases were downregulated. Succinate dehydrogenase is one of the major sources of O_2_^-^ (80). Thus, changing cell respiration to produce energy during aerobic infection could be the strategy used by ϕSan23 to control oxidative damage, while in the anaerobic infection, the strategy is not needed because the conditions of the whole environment help to decrease oxidative stress. In fact, in the anaerobic infection, the expression of thiosulfate and sulfate transport genes increased and *Salmonella* s25pp cells were probably using these compounds as electron acceptors. Moreover, the anaerobic respiration mechanism induced in anaerobiosis was nitrate reduction, the best option after oxygen for energy generation. Although the energy produced is reduced, the energy yield should be enough for viral replication. In *Enterobacter aerogenes*, ATP production decreased from 36 to 3 molecules using nitrate as an electron acceptor in anaerobiosis (81), while in *Pseudomonas stutzeri*, the production of ATP in oxidative phosphorylation only went down from 3 ATP per oxygen atom to 2 ATP per reduced nitrate (82).

A complete understanding about why infection in anaerobiosis is different from aerobiosis was not possible since many differentially expressed genes belonged to the unknown category in bacteria, and most phage genes could not be annotated. However, we did demonstrate that the infection in anaerobiosis is slower; this is evident in the one-step curve and in the transcription profile over time. We found no differential expression in the comparison at the end of the infections. One of the few differences detected in the anaerobic infection was the overexpression of the AST pathway, of which the final product is glutamate. The gene *putA* that oxidizes proline to produce glutamate was also overexpressed. Nonetheless, none of the genes related to using glutamate were differentially expressed. An accumulation of glutamate is required to maintain the steady-state potassium (K+) pool and that K+ glutamate is required for optimal growth (83). The Kdp– potassium transport system was upregulated. Why the bacterium or the phage induced this metabolic pathway only in anaerobic infection is not yet clear. Potassium is the major intracellular cation in bacteria; it plays four roles in bacteria: osmotic solute; activator of intracellular enzymes; regulator of internal pH; and second messenger (84). Even so, the *proU* system that induced betaine transport has been reported to control osmotic stress in *Salmonella* Typhimurium (85) and the genes *proV* and proW—belonging to the *proU* system—were also upregulated during anaerobic infection. This metabolic behavior could be a response to osmotic stress in anaerobic infection, but this requires further analyses.

The putative genes *rtcA* and *rtcB* were upregulated in aerobic and anaerobic infection. RtcA and RtcB are involved in the RNA repair process but their function in bacteria such as *Salmonella* or *E. coli* is not fully understood. Kurasz et al. observed that these genes in *Salmonella* Typhimurium respond to conditions such as genotoxic (mitomycin C), metabolic (nitrogen limitation), and oxidative (peroxide) stress (86). Some authors have reported that bacterial RNA is degraded during phage infection (68, 77, 87, 88). We observed that the number of bacterial transcripts decreased, while the phage transcripts increased as the infection progressed (Fig. 5C). Thus, the expression of putative RNA repair genes could control RNA degradation by the bacterium.

The appearance of bacterial resistance against phages is an important parameter in phage therapy. As a result, we evaluated the development of resistance in anaerobiosis to assess the behavior of *Salmonella* with the phage in conditions of limited oxygen, as its application is intended for anaerobic environments, i.e., phage therapy in animals or human intestines. Holguin et al. in a co-evolutionary experiment observed that variants of *Salmonella* s25pp resistant to ϕSan23 had no mutations of the CRISPR-Cas system, but in a putative prophage region and some membrane proteins (24). According to this result, we proposed that a plausible resistance mechanism used by the bacteria was the mutation of the BtuB protein, receptor of the ϕSan23 phage. But at 4 h and 24 h post-infection, the resistant variants showed no mutations in the *btuB* gene. This means that *Salmonella* s25pp is using mechanism(s) to gain resistance to the phage other than the CRISPR-Cas system and modifications in the phage receptor.

Bacteria have an arsenal of different mechanisms to resist phage attacks such as spontaneous mutations, restriction-modification systems, and adaptive immunity via the CRISPR-Cas system. Spontaneous mutations are the main mechanism because they may confer phage resistance by modifying the structure of the phage receptors, directly related to phage specificity (89). Other resistance mechanisms include the prevention of phage adsorption or phage DNA entry, targeted cleavage of phage nucleic acids, toxin/antitoxin systems, abortive infection systems or quorum sensing, and prophage-mediated mechanisms that prevent super-infection. Wang et al. demonstrated in *Salmonella* Typhimurium that different mechanisms could function together in a single strain. Their results showed that a strain can prevent phage adsorption, phage DNA entry, induce the awakening of the SOS system, and the targeting of phage nucleic acids (43). In our case, we ruled out CRISPR-Cas and modifications in the BtuB receptor among the potential resistance mechanisms. Moreover, the multiple mutations detected in the prophage of *Salmonella* s25pp in aerobic conditions by Holguin et al. suggested a potential role of the prophage in the development of resistance. So far, we have not identified the resistance mechanism(s) used by *Salmonella* s25pp against ϕSan23, nor do we know whether the mechanism(s) depends on the availability of oxygen in the medium. Nonetheless, the results here presented showed differences in the proportion of resistant isolates obtained in aerobic and anaerobic conditions. Although anaerobiosis has not been described as a factor that affects resistance mechanisms, some resistance mechanisms do seem to be affected by environmental parameters such as temperature. For example, a restriction-modification system in *Listeria monocytogenes* was described as being responsible for temperature-dependent phage resistance (90). Thus, the absence of oxygen might be responsible for a phage resistance mechanism given that some forms of resistance against phages are activated in specific environmental conditions. The transcriptomic analysis showed that the toxin/antitoxin system *dinJ-yajQ* was upregulated in anaerobic infection. *dinJ-yajQ* has not been reported as a resistance mechanism against phages although it has been associated to general stress response (91). Furthermore, in *E. coli*, five toxin/antitoxin systems, including *dinJ-yajQ*, appeared to have a bacteriostatic rather than a bactericidal effect (92) under nutritional stress. If the activation of this system in *Salmonella* s25pp in an anaerobic environment has the same bacteriostatic effect, it could explain why the infection in anaerobiosis is slower, why the RBPT decreases, and why the viral life cycle is affected. Moreover, this system could be the resistance mechanism used by these bacteria in anaerobiosis that is not too effective in preventing infection as the mechanism used in aerobiosis. Thus, in anaerobiosis, the bacteria would affect the infection by a toxin/antitoxin system, while in aerobiosis, it can use other mechanisms that are more effective in terms of survival.

We also examined the role of the BtuB protein in terms of transcription, expression, and mutational changes. In our biological model, BtuB is important as it is the receptor for ϕSan23 (24) and because in *Salmonella* sp., around 1% of its genome is related to biosynthesis or transport of cobalamin (vitamin B12) (93). BtuB is the transport protein for cobalamin, and it is regulated by the concentration of this vitamin in the cell (93). Moreover, *Salmonella* can synthesize cobalamin only in anaerobiosis (19–21, 94). We confirmed in the bacterial transcriptome without infection that the genes to synthesize B12 were upregulated only in anaerobiosis. The RT-qPCR result showed that the expression of BtuB protein increased slightly in anaerobiosis, and surprisingly, the gene presented no mutations even under the phage selective pressure. Cobalamin (vitamin B12) plays an important role in the metabolism of microorganisms; in fact, it can modulate gut microbiota (95). In the case of enteric bacteria such as *Salmonella* and *E. coli*, cobalamin acts as a coenzyme in different pathways, so it is interesting that *Salmonella* s25pp did not use the most common mechanism described to resist the phage infection (mutations in phage receptor). We speculate that *Salmonella* keeps this receptor intact because it plays an important role in its metabolism. *Salmonella* sp. has B12-dependent enzymes such as glycerol dehydratase and ethanolamine ammonia-lyase for anaerobic fermentation of glycerol, propanediol, and ethanolamine (96, 97), acetogenesis via the Wood-Ljungdahl pathway in anaerobic environments (98), aminomutases for the conversion of amino acids, and ribonucleoside diphosphate reductases for DNA synthesis that may be useful in anaerobic growth where the DNA is affected (96). Importing or synthesizing vitamin B12 is energetically costly: the biosynthesis of vitamin B12 involves at least 30 different enzymes, and the transport of exogenous B12 requires ATP (93). According to our results, the synthesis of B12 will not prevent its import during the anaerobic growth of *Salmonella* s25pp; as we observed, the amount of BtuB proteins on the cell surface increases. It is worth mentioning that increasing the amount of cobalamins in the medium represses the expression of *btuB* gene in *E. coli* (99). Finally, the regulation of *btuB* gene and genes for B12 biosynthesis requires a conservative region known as B12 elements that are highly conserved in bacteria, especially in proteobacteria, cyanobacteria, actinobacteria, and CFB group (Cytophaga, Fusobacterium, and Bacteroides) (100, 101). *Salmonella* has B12 elements, which is perhaps why generating changes in the *btuB* gene is not the first option of resistance mechanisms against phages.

### Conclusions

The absence of oxygen affects the phage-host interaction in the biological model *Salmonella* s25pp and ϕSan23. In this model, the anaerobic environment offers an interesting trade-off: the infection and viral life cycle are affected negatively (favoring the bacteria) but simultaneously diminishing the development of bacterial resistance (favoring the phages). The fact that the same condition benefits and impairs the phage and its host is a demonstration of the balance for the control of microbial populations and their coexistence in nature. Given the existence of many anoxic environments (sediments, deep seas, animal intestines, wounds), reduced burst sizes per bacterial cell infected could be a common trend *in vivo*, making mandatory the consideration of oxygen concentration for phage therapy and ecological studies. It is also an invitation to explore the physiological significance of oxygen availability for phage therapy.

## SUPPLEMENTAL MATERIAL

Data Set S1 aem.01491-23-s0001.xlsx Data set for transcriptomic analysis

Fig. S1 to S9 and Tables S1 and S2 aem.01491-23-s0002.pdf Added complementary figures and tables for Western blot assays, sequencing, and transcriptomic analysis.

ASM does not own the copyrights to Supplemental Material that may be linked to, or accessed through, an article. The authors have granted ASM a non-exclusive, world-wide license to publish the Supplemental Material files. Please contact the corresponding author directly for reuse.
