# Supplementary material for: Anaerobiosis, a neglected factor in phage-bacteria interactions
Source: Appl Environ Microbiol. 2023 Nov 15;89(12):e01491-23. doi: 10.1128/aem.01491-23 (PMC10734468; doi:10.1128/aem.01491-23)
Supplement: Fig. S1 to S9 and Tables S1 and S2 — Added complementary figures and tables for Western blot assays, sequencing, and transcriptomic analysis. [file aem.01491-23-s0002.pdf]

Anaerobiosis, a neglected factor in phage-bacteria interaction

Santiago Hernández Villamizar[1], Luis A. Chica Cárdenas[1], Laura T. Morales Mancera[1], Martha J. Vives Florez[1]

1. **Department of Biological Sciences, Universidad de los Andes, Bogotá, Colombia**

Supplementary material

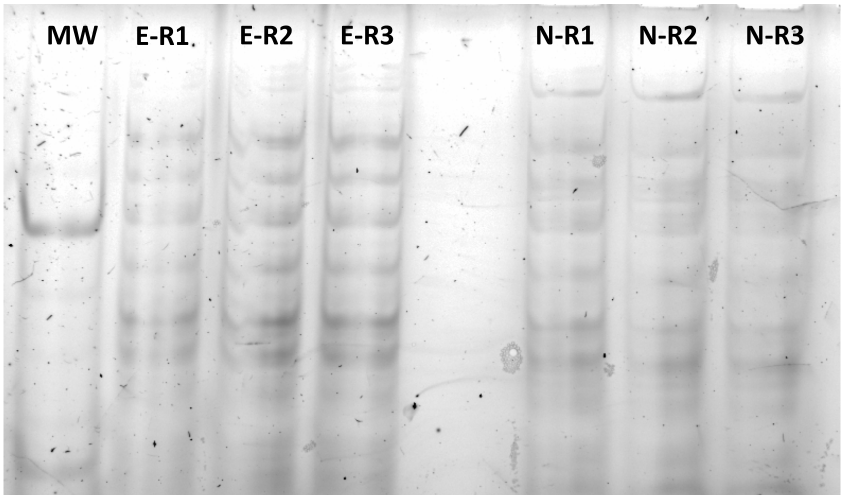

Figure S1. Gel protein to detect FtsZ protein. E: Aerobiosis, N: Anaerobiosis, MW: Molecular weight marker

Table S1. Normalization and relative quantification parameters to FtsZ protein in Western blot assay. Relative quantification was calculated respect to aerobic condition signal.

Sample	Normalization Factor	Relative quantification rep.1	Relative quantification rep.2	Relative quantification rep.3
Anaerobic rep. 1	0.670871	1.475763	0.555707	0.615367
Anaerobic rep. 2	0.71414	0.675866	0.254501	0.281824
Anaerobic rep. 3	0.738999	0.563872	0.212329	0.235125

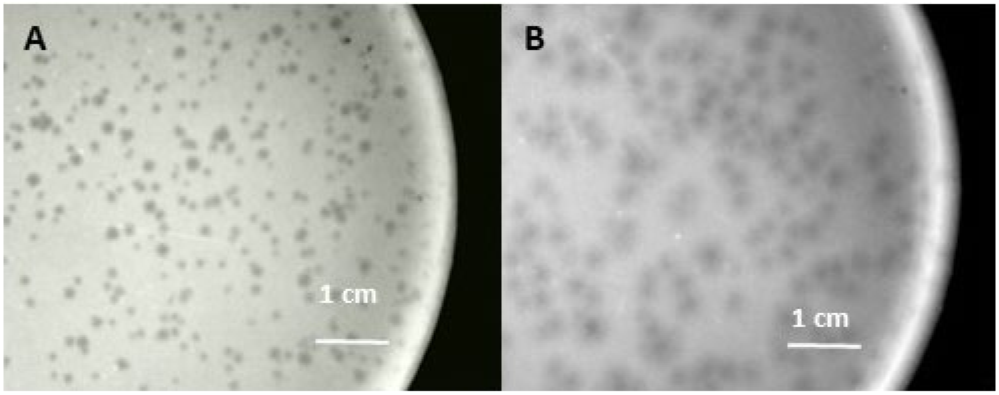

Figure S2. Plaque morphology of phage ϕSan23 under aerobic and anaerobic conditions. Left: aerobic, right: anaerobic (atmosphere of 99,7% nitrogen, Coy chamber (COY Laboratory Products®)).

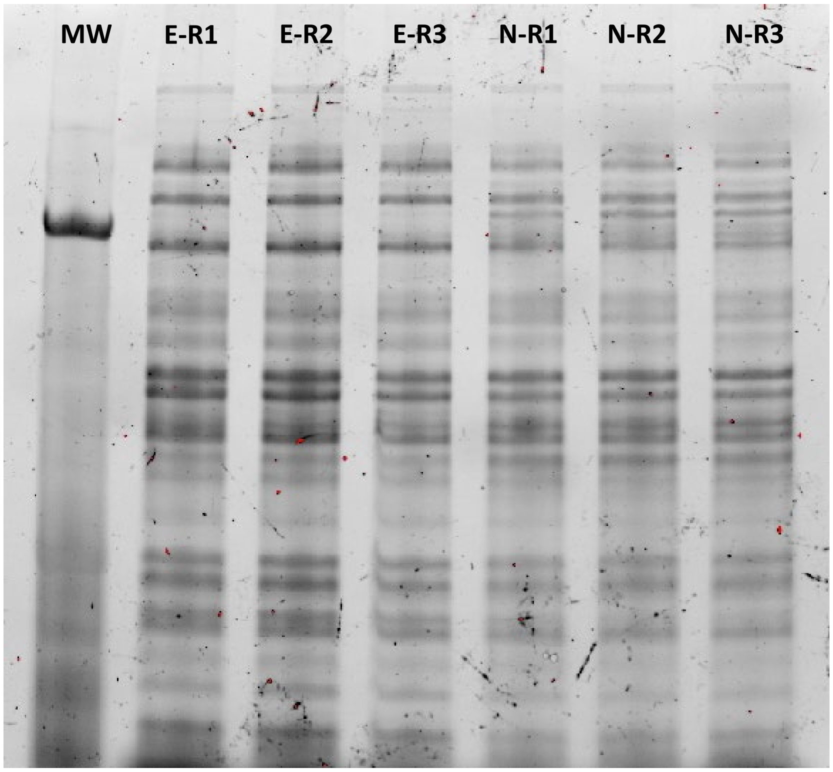

Figure S3. Gel protein to detect BtuB protein. E: Aerobiosis, N: Anaerobiosis, MW: Molecular weight marker

Table S2. Normalization and relative quantification parameters to BtuB protein in Western blot assay. Relative quantification was calculated respect to aerobic condition signal.

Sample	Normalization Factor	Relative quantification rep.1	Relative quantification rep.2	Relative quantification rep.3
Anaerobic rep. 1	1.323288	0.856853	0.673542	0.978386
Anaerobic rep. 2	1.312513	1.391248	1.09361	1.588577
Anaerobic rep. 3	1.313573	2.458188	1.932294	2.806848

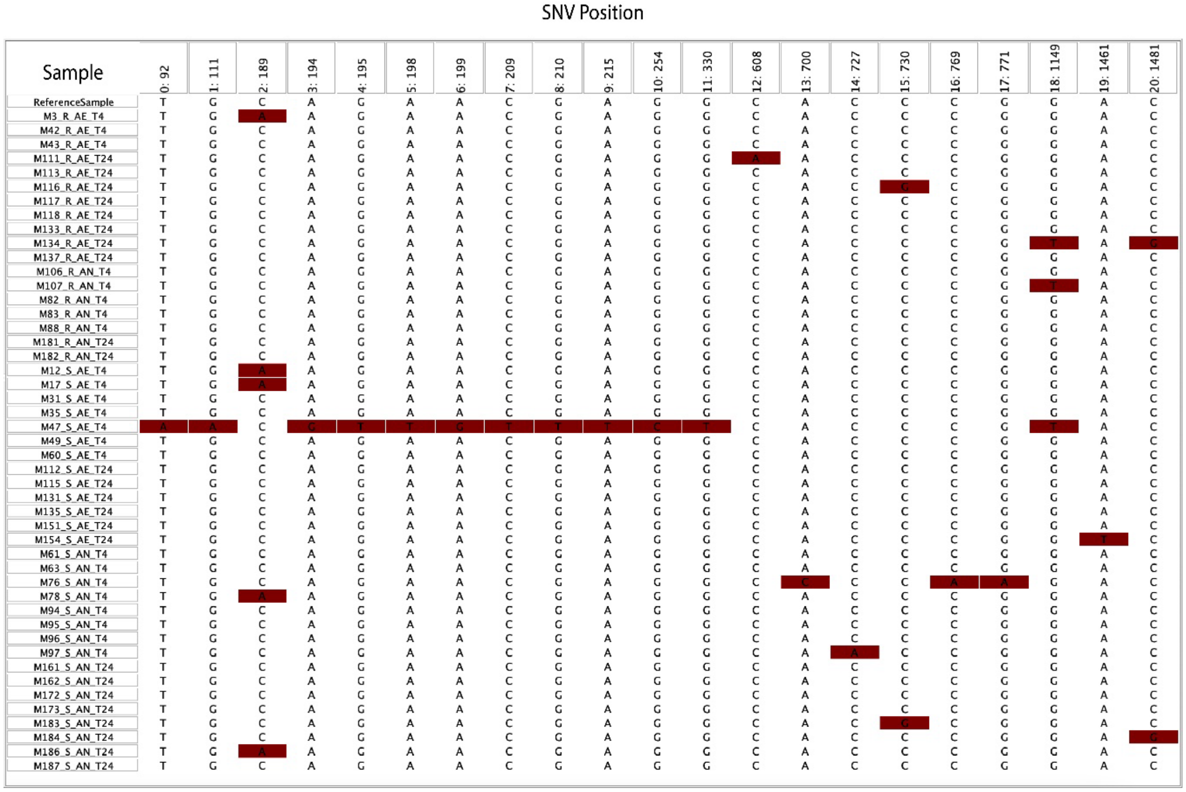

Figure S4. SNVs found in each sample after comparing against the reference (first row). SNVs are organized by their local position on the gene. SNVs found at each position confirm that SNVs are randomly distributed throughout the samples, regardless of the experimental variables.

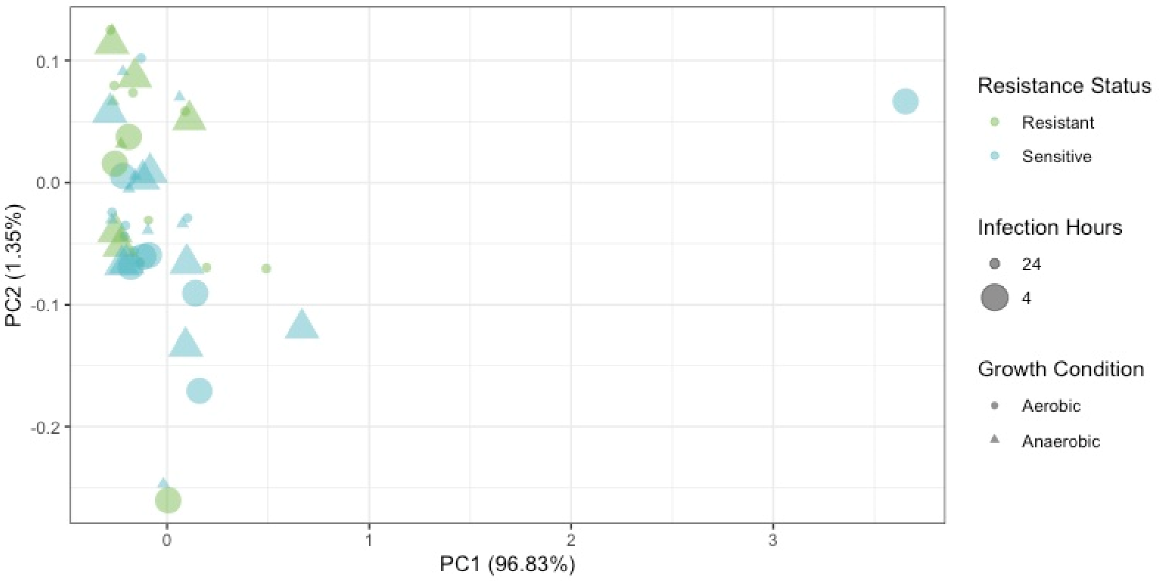

Figure S5. PCA representing the dispersion of the samples based on SNVs distribution. Samples are differentiated by their growth condition (Aerobic, Anaerobic), their resistance against phage infection and the time after phage inoculation. Since most of the sample dispersion is related to the PC2, in which a minimum portion of the variance is explained, we can infer a random pattern in the presence and location of SNVs on the samples, leading to the assumption that the different conditions tested have no influence on single nucleotide changes in *btuB* gene. Even though one sample is clearly different to the rest, the lack of relatedness with other samples associated to the same conditions suppose an association to technical factors, rather than a biological response to the testing condition.

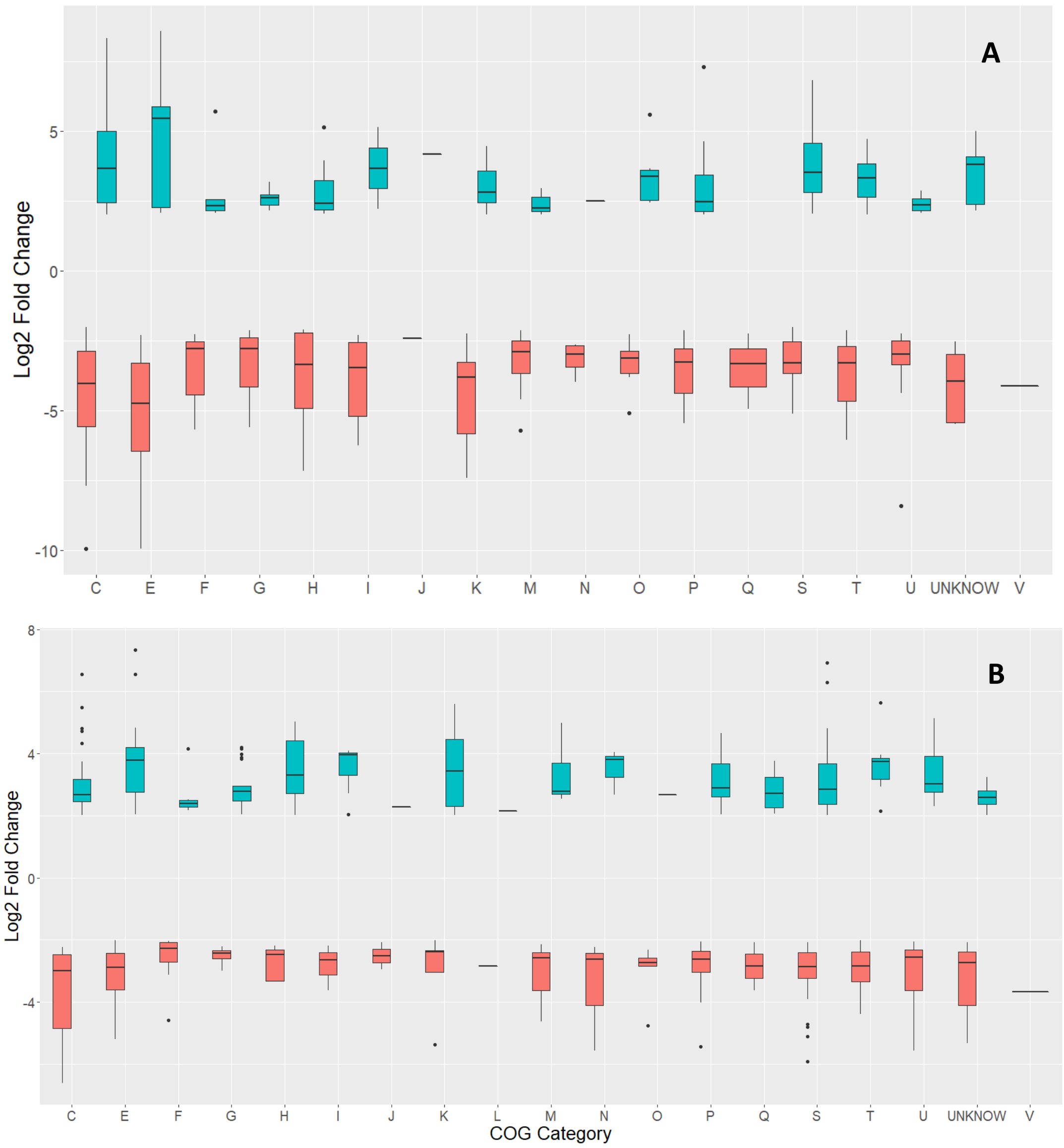

Figure S6. Log2 Fold Change of bacterial genes in anaerobic condition by COG category. A: Without infection, B: With infection. Blue: Upregulated, red: Downregulated. C: Energy production and conversion, E: Amino acids transport and metabolism, F: Nucleotide transport and metabolism, G: Carbohydrate transport and metabolism, H: Coenzyme transport and metabolism, I: Lipid transport and metabolism, J: Translation, ribosomal structure and biogenesis, K: Transcription, L: Replication, recombination and repair, M: Cell wall/membrane/envelope biogenesis, N: Cell motility, O: Post-translational modification, protein turnover, chaperones, P: Inorganic ion transport and metabolism, Q: Secondary metabolites biosynthesis, transport and catabolism, S: Function unknow, T: Signal transduction mechanism, U: Intracellular trafficking, secretion, and vesicular transport, V: Defenses mechanism, Unknow: without COG category. Genes with p-value adjustment <0.05.

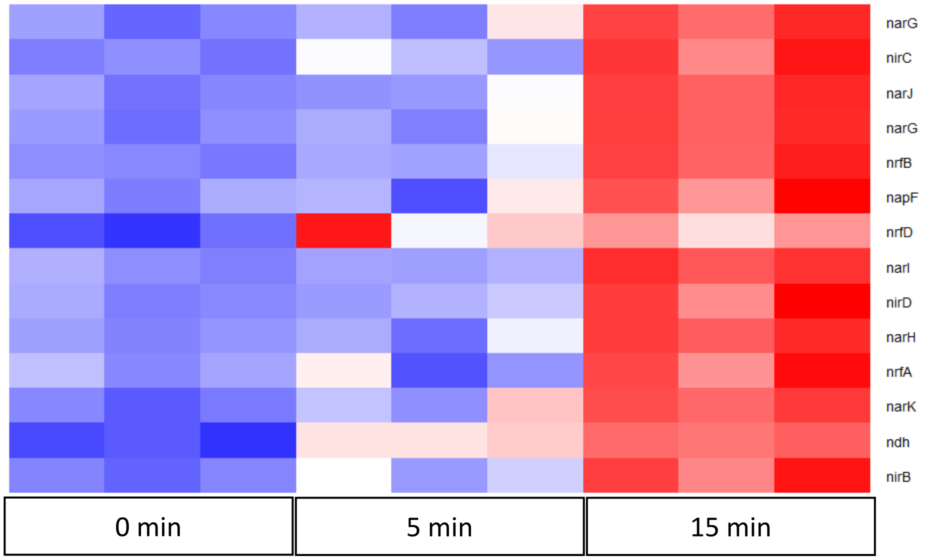

Figure S7. Bacterial nitrate reduction genes differentially expressed (-2 Log2 > Fold Change, 2 < Fold Change, p-value adjustment < 0.05) during phage infection in aerobic condition. Red: Upregulated, blue: Downregulated.

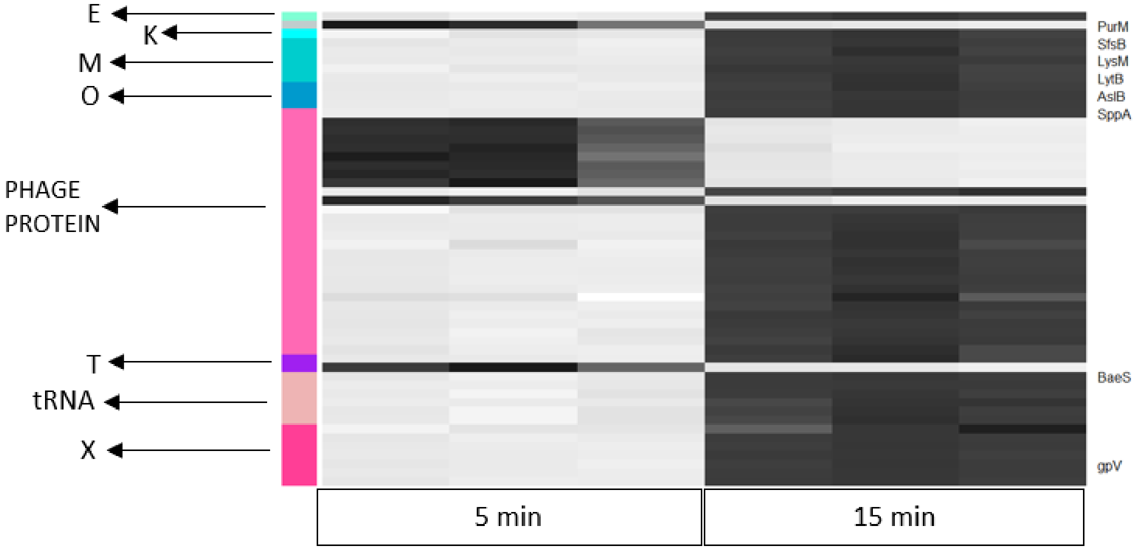

Figure S8. Phage genes differentially expressed (-2 > Fold Change, 2 < Fold Change, p-value adjustment < 0.05) during phage infection in aerobic condition in descending order by COG categories. E: Amino acids transport and metabolism, F: Nucleotide transport and metabolism, H: Coenzyme transport and metabolism, K: Transcription, M: Cell wall/membrane/envelope biogenesis, O: Post-translational modification, protein turnover, chaperones, Phage protein, T: Signal transduction mechanism, tRNA: tRNA genes, X: Mobilome (Prophages, transposons). Black: Upregulated, white: Downregulated

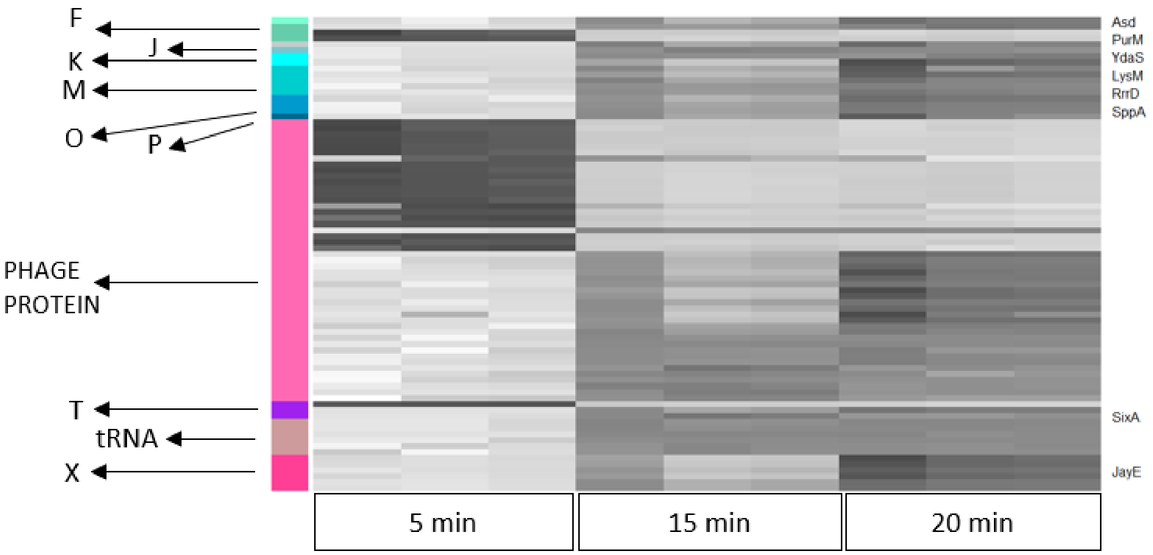

Figure S9. Phage genes differentially expressed (-2.5 > Fold Change, 2.5 < Fold Change, p-value adjustment < 0.05) during phage infection in anaerobic condition in descending order by COG categories. E: Amino acids transport and metabolism, F: Nucleotide transport and metabolism, H: Coenzyme transport and metabolism, J: Translation, K: Transcription, M: Cell wall/membrane/envelope biogenesis, O: Post-translational modification, protein turnover, chaperones, P: Inorganic ion transport and metabolism, Phage protein, T: Signal transduction mechanism, tRNA: tRNA genes, X: Mobilome (Prophages, transposons). Black: Upregulated, white: Downregulated
